# Projected climate change could expand the geographical suitability of *Acacia dealbata* into new temperate areas

**DOI:** 10.1007/s10661-026-15951-5

**Published:** 2026-09-29

**Authors:** Pedro Lago-González, Carolina Acuña-Alonso, Xana Álvarez

**Affiliations:** Hydroforestry Geomodeling Research Group, School of Forestry Engineering, Universidade de Vigo, Campus A Xunqueira S/N, 36005 Pontevedra, Spain

**Keywords:** Mimosa, Australian acacia, Invasive alien species, Species distribution, Climatic profile, Future scenarios

## Abstract

*Acacia dealbata* (Link) is enlisted as an invasive alien species in several regions where it was introduced. The invasiveness of the species relies on its resilience to disturbances such as bushfires or water depletion, humans favouring their introduction, or their ability for nitrification of soils. Understanding the potential spread of the species and their climatic niche is of great interest given their ease to invade new environments. In this research, we aimed to predict the potential distribution of *A. dealbata* in *Quercus robur* L. forests. Maximum entropy models were developed to simulate the past, present, and future climatic niche suitability for *A. dealbata* and *Q. robur*. The potential distributions of the species in several periods of study were compared through Schoener’s and Hellinger’s indices, and climatic profiles were developed as result of variable contributions and response curves. The models performed with great accuracy. The variables accounting the most for the distribution of the species were related to temperature. The main hotspot of niche overlap between *Q. robur* and *Acacia dealbata* was Europe. Our results showed that even if both species share an important extent of area worldwide, their suitable climatic niches do not completely overlap.

**Supplementary Information:**

The online version contains supplementary material available at https://doi.org/10.1007/s10661-026-15951-5.

## Introduction

Australian *Acacia* is a genus of leguminous woody plants (~ 1000 species) originating in continental Australia and the neighbouring islands, such as the Island of Tasmania (Lorenzo et al., 2010). Across this region, a wide range of climate types can be found according to the Köppen-Geiger classification (Beck et al., 2018). The climatology of the coastal regions of south-western and south-eastern Australia is similar to that of western and southern Europe, where temperate and Mediterranean climates predominate (Beck et al., 2018). Some species of *Acacia* were introduced to Europe in the late 1700 s as an ornamental plant (Kull & Rangan, 2008; Sheppard et al., 2006) as well as for economic reasons, primarily as sources of fuelwood and construction wood (Kull & Rangan, 2008). They have become particularly successful in the South-West and Mediterranean regions of Europe (Marchante et al., 2023). As a result of human-mediated introduction, many *Acacia* species have spread into new environmental niches, displacing native species and invading these areas (Wilson et al., 2011). In fact, some *Acacia* species have been classified as invasive alien species (IAS) in some countries, such as Spain (Ministerio para la Transición Ecológica y el Reto Demográfico, MITECO, 2007) or Portugal (Ambiente e Transição Energética, 2019). In addition, *Acacia saligna* (Labill.) H.l. Wendl. is enlisted in the invasive alien species of Union concern of the European Union Regulation No. 1143/2014 (EU, 2022). Italy (Galasso et al., 2018; Minissale et al., 2023) and France (González-Muñoz et al., 2012; Marchante et al., 2023) are also hotspots for the proliferation of some species of *Acacia*, although these countries do not have any specific legislation categorising the genus as invasive. Some of the major invasive *Acacia* species in Europe include *Acacia dealbata*, *Acacia melanoxylon* R. Br., *Acacia longifolia* (Andrews) Willd., *Acacia retinoides* Schltdl., and *A. saligna* (Lorenzo et al., 2010).

There are several factors explaining the spread of Australian acacias in places where they are non-native. In the first place, humans have played a key role, not only introducing and disseminating them to new environments, but also clearing the native competing vegetation (Richardson et al., 2011). Moreover, their rapid growth favours their competition for sunlight over some of the native species (Morris et al., 2011). Because of their extensive root systems, they also lead to water depletion in the ecosystem where they establish, implying a reduction in the availability of such resources for the native species (Morris et al., 2011). Furthermore, they are generalists in terms of nutrient availability (Morris et al., 2011). *Acacia* species show greater resilience to disturbance events such as anthropogenic soil disturbance, forest fires, or higher atmospheric carbon dioxide concentrations (Lorenzo et al., 2010; Souza-Alonso et al., 2017). Therefore, climate change has the potential to worsen the effects on local biodiversity that such invasions may produce.

*Acacia* species exhibit invasive potential in areas where *Quercus* spp. occurs, either naturally (Europe); naturalised as in Australia (Lohr & Keighery, 2016); or as an IAS (South Africa) (Gildenhuys et al., 2024). This suggests that acacias and oaks may occupy similar climatic niches and therefore may compete for resources in regions where they co-occur. Competition between Australian acacia species and native species on the Northern Hemisphere (NH) temperate rainforests has been studied before. González-Muñoz et al. (2012) reported that *A. dealbata* produces a deterioration of soil quality in *Q. robur* forests in north-western (NW) Spain, implying a potential threat to the native species. Moreover, González-Muñoz et al. (2011) stated that *A. dealbata* and *Eucalyptus globulus* have some advantageous traits over native *Q. pyrenaica* and *Pinus pinaster*. These traits were their resprouting ability, the allelopathic leachates and, specifically for *A. dealbata*, its nitrogen-fixation ability. Also, Martín-Forés et al. (2023) linked the invasiveness of Australian acacias to functional trait strategies such as maximising plant height, allowing them to outcompete other species in the competition for sunlight.

Potential distribution of invasive species is of great interest and has been widely studied. Species distribution models (SDMs) are a common approach for addressing this question. Regarding Australian *Acacia* species, there is an extensive body of literature on the matter, with a special focus on *A. dealbata*, *A. longifolia*, *A. saligna*, and *A. melanoxylon.* These studies have been conducted mostly in regions where these species are classified as IAS, such as the NW of the Iberian Peninsula, South Africa, or Chile. In the NW Iberian Peninsula, a common approach has been to use ensembles of either all or some of these models to predict potential distribution of *A. dealbata*, *A. longifolia*, and *A. melanoxylon*. Another region where SDM studies on Australian acacias have been widely conducted is in South Africa, where they focus mainly on *A. dealbata* and *A. saligna*. Other studies include distribution of *A. saligna* in Sardinia (Italy) or Egypt. Even if the majority of these studies are conducted at the local or statal level, there are some examples of global-scale SDM studies as well (Booth, 2024; Higgins et al., 2020).

These models usually achieve high performance for the prediction of species distribution. In this context, the main objective of this work is to assess the effects of climate change on the global distribution of *A. dealbata*, which has been historically introduced to new areas. In this study, we propose an SDM-based methodology to evaluate the changes in the past, present, and future potential distribution of *Acacia* worldwide under the influence of climate change, and the effects these changes could have on oak (*Quercus robur* L.) forests in the NH. This, with the specific objectives of (i) understanding the factors that led to the invasion of *Acacia* in some areas such as Europe; (ii) assessing the changes in the climatic suitability for the species in these new areas; and (iii) evaluating the potential co-occurrence existence with other species such as *Q. robur*, which is a representative species of one of the habitats most sensitive to invasion of *Acacia*. We hypothesise that, owing to its invasive characteristics and broader ecological tolerance, *A. dealbata* will be less negatively affected by climate change than the control species (*Q. robur*).

## Materials and methods

### Data acquisition and preprocessing

Climate data was used to characterise the climatology worldwide in two differentiated periods: the former covering from 1963 to 1992, representative of the recent past climatic period; and the latter from 1993 to 2023, as a depiction of the current climatic stage. This data was retrieved from the National Centers for Environmental Prediction/National Center for Atmospheric Research (NCEP/NCAR) reanalysis 1 project (Kalnay et al., 1996), provided by the National Oceanic and Atmospheric Administration Physical Sciences Laboratory (NOAA PSL) Boulder, CO, USA (https://psl.noaa.gov/). For future scenarios, projections of the Coupled Model Intercomparison Project 6 (CMIP6) (doi.org/https://doi.org/10.24381/cds.c866074c) for the Community Earth System Model 2 (CESM2) (Danabasoglu, 2019) were obtained from the Copernicus Climate Data Store (CDS) catalogue (2021) (accessed on 2024-12−10), for the shared socioeconomical pathways: SSP1-2.6, SSP2-4.5, SSP3-7.0, and SSP5-8.5.

Historical daily average, maximum, and minimum air temperature at 2 m above ground level (m.a.g.l.) (K) were obtained for the period between 1963 and 2023, covering from 180° W to 180° E and from 90° N to 90° S, with a spatial resolution of 2° × 2°, which was downscaled through linear interpolation to a 0.5° × 0.5 grid resolution. Projected data from 2024 to 2053 was obtained for each SSP and resampled to the same grid. The same pipeline was applied to daily average precipitation (obtained as precipitation rate in units of kilogramme per square metre and per second (kg m^−2^ s^−1^) and converted to millimetres per square metre (mm)). Specific humidity (*q*) was also supplied, in units of kilogrammes of water vapour per kilogramme of moist air, converted to g kg^−1^. Finally, the downwards solar radiation (*I*) was acquired from the same sources, in W m^−2^. For each period, the average of specific humidity and radiation was calculated. Furthermore, the annual range (as the difference between the maximum and the minimum month averages) was obtained along with the seasonality, as 100 times the standard deviation (Eq. 1).

1 $${x}_{\mathrm{s}\mathrm{e}\mathrm{a}\mathrm{s}\mathrm{o}\mathrm{n}}=100 \sigma$$

where *x*_season_ represents the seasonality of the variable *x*, and *σ* is the standard deviation of its monthly average. These were obtained for the temperature as well, in addition to the mean daily thermal range and the isothermality, which is the portion of the daily range that explains the annual range (Eq. 2), and has been linked to species distribution (Chen et al., 2023; Zhong et al., 2022).

2 $$Iso=\frac{{({T}_{\mathrm{m}\mathrm{a}\mathrm{x}}-{T}_{\mathrm{m}\mathrm{i}\mathrm{n}})}_{\mathrm{d}\mathrm{a}\mathrm{i}\mathrm{l}\mathrm{y}}}{{(\left|{T}_{\mathrm{m}\mathrm{a}\mathrm{x}}\right|-\left|{T}_{\mathrm{m}\mathrm{i}\mathrm{n}}\right|)}_{\mathrm{a}\mathrm{n}\mathrm{n}\mathrm{u}\mathrm{a}\mathrm{l}}}$$

where *Iso* represents the isothermality, *T*_max_ and *T*_min_ are the daily maximum and minimum temperature, respectively, and |*T*_max_| and |*T*_min_| are the maximum and minimum monthly averages. On the other hand, the processing of the precipitation series differed from the other climate variables. First, the average annual accumulated precipitation was calculated. Then, the annual minimum and maximum precipitation were obtained as the accumulated precipitation of the driest and the wettest months, and the annual coefficient of variation of the monthly accumulated was taken as the seasonality of precipitation.

Using the climate values of the variables during the period from 1991 to 2020 as the baseline reference for historical data, and 2021–2050 for projections data, annual anomalies of the mean temperature, radiation and humidity; and accumulated precipitation were obtained. A spatial empirical orthogonal function (EOF) analysis (Lorenz, 1956), with varimax rotation (Kaiser, 1958), was carried for each variable. These are regarded as proxies of the climatic interannual variation, which may or may not be representative of variations resulting from climate change.

Considering the potential collinearity, we performed a filtering on the parameters based on their physical meaning. Therefore, for temperature, radiation, and humidity, we selected the climate mean, representing the baseline signal; the annual range and seasonality, representing the periodic variability at different time windows; and the components of the EOF analysis that follow the Kaiser rule (eigenvalues greater than 1), representing the interannual climate variability. In addition, the isothermality and daily range were used as inputs. Regarding precipitation, the climate cumulative annual was the baseline; seasonality and monthly maxima and minima represented the periodic variability; and the EOFs represented the interannual variability. No further preprocessing was applied to the data, reducing collinearity while maintaining the physical interpretability of the inputs.

Data on plant species occurrence was retrieved from the Global Biodiversity Information Facility (GBIF, https://www.gbif.org/). For each climatic period, data on presence of *A. dealbata* was collected*.* For comparison with a low-invasive-potential species, data of presence of *Q. robur* was provided*.* Duplicate and offshore records were removed and then spatial thinning (Aiello-Lammens et al., 2015) for a 25-km radius was applied to prevent biases from the geographically uneven sampling. The remaining points were downsampled, randomly selecting 25% of the presence points. Based on the species presence locations, a set of background points was generated for each species and period. To avoid sampling bias, a buffer zone was created, comprising 150 km from the outermost presence points in a cluster. A random set of background points 10 times larger than the presence points was generated, weighed by Nelson’s (2008) global map of accessibility. This was done to replicate the sampling bias in the presence data.

### Climatic profile and maximum entropy model

The *Acacia* species assessed in this study is native to the temperate areas of Australia. Although the main climate on the country is arid (BWh, BSh), covering 78% of its area (Beck et al., 2018; Hall et al., 2022); the eastern coast, along with the island of Tasmania, presents temperate climates, which account for a total of 14% of its area (Hall et al., 2022). Also, the south-western coast climate type is Mediterranean of dry summer, either hot or warm (Csa, Csb), while the eastern does not, in general, register a dry season, with hot and warm summers (Cfa, Cfb). Regarding the island of Tasmania, the predominant climate is the Cfb; however, the most inland part is Oceanic with cold summers (Cfc). Some eastern mountainous regions are of cold climate with warm summers and no dry season (Dfb). These coastal regions are where most species of *Acacia* naturally occur (Adiamo et al., 2020). These are also the type of climates where *Q. robur* forests naturally occur in the NH, especially in Europe and western Asia (Degen et al., 2021).

To assess the invasion status of *Acacia* in temperate forests, a maximum entropy model (MaxEnt) was applied to predict the climatic suitability and potential niche of both *A. dealbata* and *Q. robur*. The basis of this model is to estimate a density distribution function such that the entropy is maximum, meaning that the distribution is the most uniform possible, subject to the empirical data (Jaynes, 1957). This kind of model has been widely applied in the prediction of species distribution, given its ease of interpretation and generally accurate results. The models were trained using a fivefold cross-validation and random sampling for testing. Their performance was evaluated with metrics such as the Area Under Curve (AUC) and the Receiver Operator Curve (ROC), the regularised and unregularised training and test gains.

The MaxEnt model was used to calculate probabilities of favourable conditions for each species and period, depending on climatic variables. The differences in the predicted distributions between periods were assessed to understand the changes in the spread of the species. Additionally, the contribution, permutation importance and response curves of each variable were evaluated to assess their influence on the results of the model. This was supplemented with a Jackknife test (Miller, 1974). This is a form of cross-validation that addresses the performance of the model by iteratively isolating and excluding each variable separately to quantify its influence in the results (Rogers et al., 2020). Furthermore, a statistical comparison of variable means using a two-tailed *t*-test with a 95% confidence level was used to assess the significant differences of the climatology in the areas where each species occurs.

### Schoener’s D and Hellinger’s I

Schoener’s D (Schoener, 1968) and Hellinger’s I (Warren et al., 2008) are quantifiers of the spatial overlap of the probability densities of two species. Both metrics quantify the relationship between the probabilities of occurrence of the two species across grid cells (Eqs. 3 and 4). These indices were calculated for all climatic variables, and the differences between periods were compared to assess the evolution in the distribution overlap of the studied species.

3 $$D=1-\frac{1}{2}\sum_{i}\left|{p}_{a,i}-{p}_{q,i}\right|$$

4 $$I=1-\frac{1}{2}H$$

where *D* and *I* are respectively the Schoener and Hellinger indices; *p*_*a,i*_ and *p*_*q,i*_ are the probabilities of finding *A. dealbata* and *Q. robur* in the *i* grid cell; and *H* is Hellinger distance (Warren et al., 2008), as expressed in Eq. 5.

5 $$H=\sqrt{\sum_{\mathrm{i}}{\left(\sqrt{{p}_{a,i}}-\sqrt{{p}_{q,i}}\right)}^{2}}$$

## Results

### Maximum entropy model performance

The MaxEnt models generally showed accurate results, with high training and test AUC (Table 1). The test AUC generally obtained greater deviation than the training. However, the gap between the regularised and unregularised training gains is higher in the *Acacia* model of the period 1963–1993 (difference of 0.381). Also, the uncertainty in the test gain for this model was higher than in the others. In comparison, all the other models resulted in more moderate gaps between regularised and unregularised train gains. Both *Q. robur* models obtained 0.169 and 0.109 for the past and present models, respectively, with test gain uncertainties similarly moderate. On the other hand, the 1993–2023 *Acacia* model gap between regularised and unregularised training gains was 0.121, but the test gain uncertainty was the order of 10 times lower than that for the oak models. For both species, the entropy was higher in the 1993–2023 period than in the 1963–1993. Our results are in line with other studies for *A. dealbata*, which usually perform with an AUC over 0.90 (Bustamante et al., 2022; César de Sá et al., 2019; Fernandes et al., 2019; Hirsch et al., 2017; Langdon et al., 2019; Monteiro et al., 2017; Silva et al., 2023).

**Table 1:** Metrics of the performance of the maximum entropy models for *A. dealbata* and *Q. robur* in the 1963–1993 and 1993–2023 periods

Statistic	*A. dealbata*		*Q. robur*	
	1963–1993	1993–2023	1963–1993	1993–2023
Training samples	49	291	74	200
Test samples	12	73	18	50
Background points	738	4189	1045	2831
AUC train	0.950 ± 0.003	0.937 ± 0.001	0.920 ± 0.028	0.921 ± 0.002
AUC test	0.913 ± 0.042	0.927 ± 0.005	0.900 ± 0.010	0.910 ± 0.016
Reg. training gain	1.701 ± 0.063	1.650 ± 0.013	1.350 ± 0.028	1.422 ± 0.029
Unreg. training gain	2.082 ± 0.063	1.771 ± 0.011	1.519 ± 0.028	1.531 ± 0.029
Test gain	1.589 ± 0.472	1.640 ± 0.051	1.300 ± 0.120	1.401 ± 0.178
Entropy	4.905 ± 0.061	6.697 ± 0.014	5.611 ± 0.022	6.532 ± 0.030

*Reg.* regularised, *Unreg* unregularised

According to the MaxEnt model, *A. dealbata* is predicted to have a highly suitable niche in the areas where it is native, including the south-eastern and south-western coasts of Australia, as well as on the island of Tasmania (Fig. S1). The climate types in these areas are predominantly temperate and Mediterranean, which may explain the successful establishment of *Acacia* on the climatically similar regions of the Mediterranean and south-western Europe, where our results show a high suitability. At present, other areas with high predicted suitability include South Africa, New Zealand, some areas in Chile, and the Pacific Coast of North America (Fig. S1). All these regions share temperate climatic conditions that appear favourable for the establishment of *A. dealbata*. In contrast, *Q. robur* is likely to find its niche mainly in Europe, extending to regions with continental climates (Fig. S2).

### Analysis of variable contributions, response curves

**Fig. 1:**
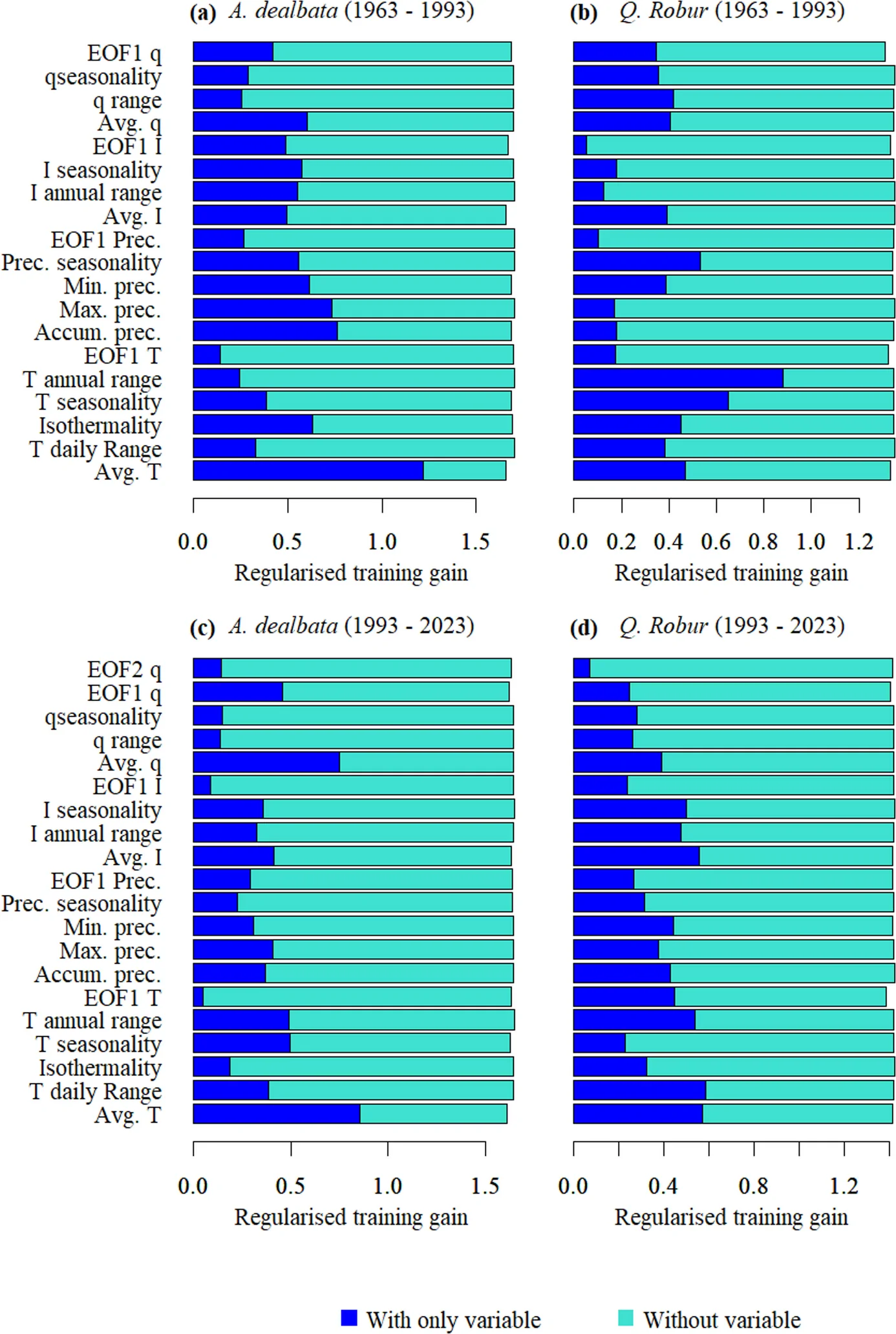
Jackknife test for the training gain. **a** *A. dealbata* model for the 1963–1993 period, **b** *Q. robur* model for the 1963–1993 period, **c** *A. dealbata* model for the 1993–2023 period, and **d** *Q. robur* model for the 1993–2023 period. *T* is temperature, *prec*. is precipitation, *I* is solar radiation and *q* is specific humidity. *Min*. is the minimum, *Max*. is the maximum, *Avg*. is the average, *Accum. is the accumulated, and EOF is the Empirical Orthogonal Function applied to the variable*

For the *Acacia* models, one of the most influential variables was average temperature, contributing 42% and 25% respectively in the past and present models. In the former, this variable was by far the most important, followed by the minimum precipitation (contributing 18%) and the radiation seasonality (12%). In the 1993–2023 period, the most influential variable was the temperature seasonality (28%). However, regarding the permutation importance, the highest was obtained for the average temperature (39%), followed by the seasonality (30%). The Jackknife test (Fig. 1) generally agreed with this, giving average temperature the greatest training gain in both periods. In the 1993–2023 period, the average specific humidity presented a comparable training gain when used alone.

In comparison, the temperature annual range was the most important variable contributing to both oak models. It obtained a contribution importance of 60% on the 1963–1993 period (permutation importance of 16%), exceeding all the other input variables. In this period, the permutation importance was higher for the specific humidity first EOF (32%), while the temperature annual range and the average obtained a similar value (17% and 15% respectively). On the 1993–2023 model, the temperature annual range contributed 27%, followed by the minimum precipitation and the radiation range, both contributing 19%. For this period, the permutation importance was highest for the minimum precipitation (15%), followed by the temperature average and first EOF (14% both), and then average specific humidity and temperature annual range (11% both). In contrast to the *Acacia* models, the Jackknife test for the oak models did not identify a single dominant predictor, particularly for the 1993–2023 period. Here, the Jackknife test on the oak model showed similar training gains across the humidity variables (average, seasonality, and annual range) as well as for the temperature (average and daily and annual ranges). In comparison, the 1963–1993 oak model showed a higher training gain using only temperature annual range, compared to other variables.

**Fig. 2:**
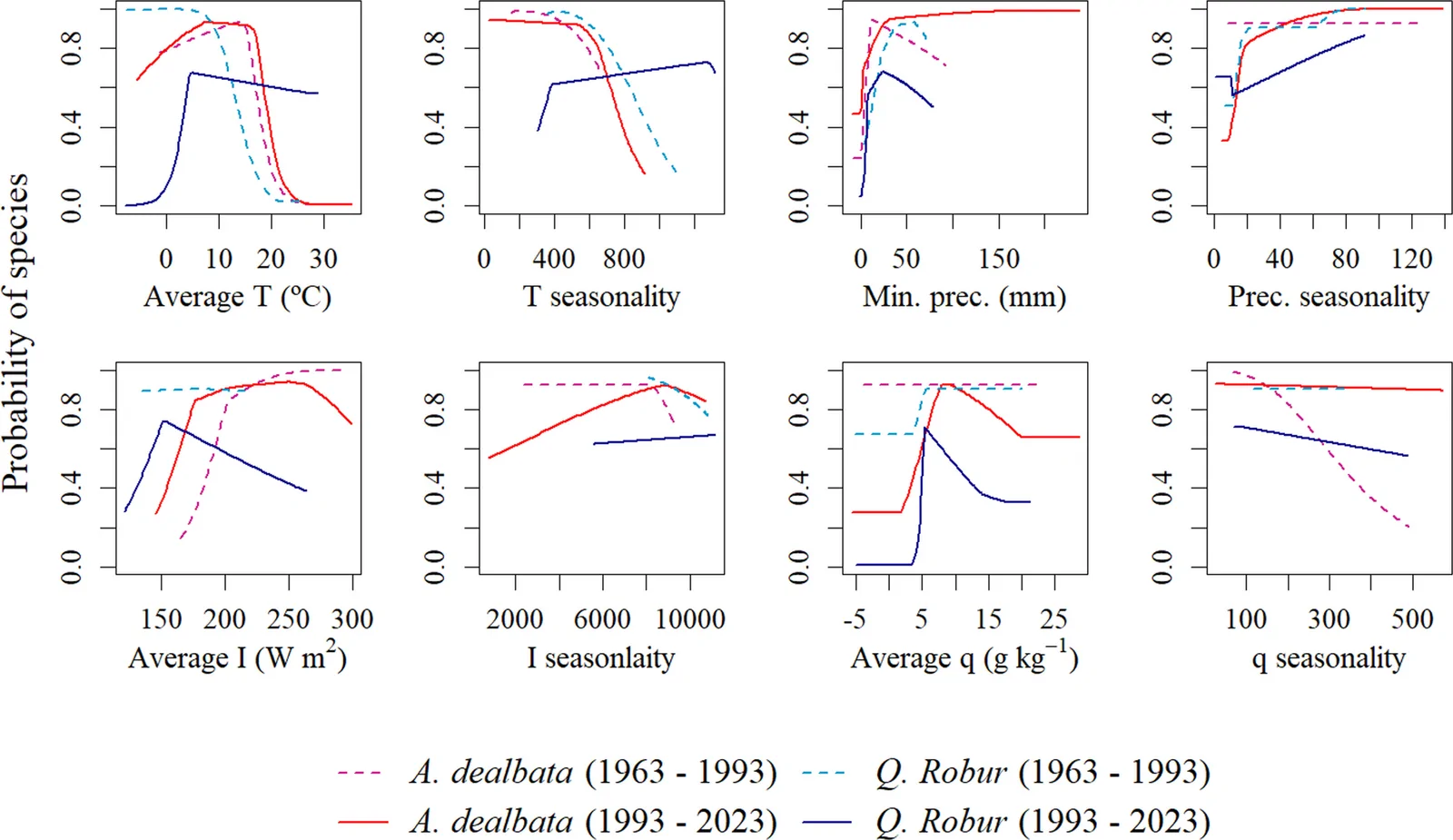
Response curves for the most important variables of the distribution models. *T*: temperature; *prec*.: precipitation; *I*: solar radiation; *q*: specific humidity

Response curves for some of the most important variables are represented in Fig. 2. For both *Acacia* models, the suitability increases with the average temperature to 13–20 °C, dropping to near 0 values at ~ 25 °C. The oak model, on the other hand, showed different patterns in the two periods. The 1963–1993 oak model showed a logistic decline of the suitability with temperature, decreasing from ~ 13 °C. The 1993–2023 model, nonetheless, shows an increase in the suitability, starting at ~ 2 °C and reaching a maximum at ~ 8 °C, where it starts gradually decreasing, following a linear trend. Regarding temperature seasonality, both *Acacia* models and the 1963–1993 oak model show a similar curve, keeping the probability of occurrence more or less constant at ~ 600, from where a quick decrease starts. The 1993–2023 oak model depicts an increase on the species suitability with the temperature seasonality.

*Acacia* models showed a quick increase of climate niche suitability with minimum precipitation, reaching a point of inflection at ~ 25 mm, from where the models behaved differently. The 1993–2023 model showed a stabilisation, while the 1963–1993 showed a linear decrease. The *Acacia* and oak models showed a similar behaviour with the latter showing a smoother curve, and with a substantially lower maximum for the 1993–2023 period. The precipitation seasonality shows a logarithmic curve for the past oak and the present *Acacia* models. The past *Acacia* model shows a very small decreasing linear trend, while the present oak model depicts a stepped decrease at low seasonality values, to then show an increasing linear trend (from ~ 10).

Both *Acacia* models increased the species suitability with average radiation at 150–200 W m^−2^. However, while the 1963–1993 model shows a logistic growth, the 1993–2023 model resulted in a bell-shaped function, starting to decrease at ~ 280 W m^−2^. The 1963–1993 oak model was largely independent on the average radiation, while the 1993–2023 model depicted a steep increase to ~ 150 W m^−2^ to quickly start dropping probability from there on. The past *Acacia* model shows a constant value with the radiation seasonality until it reaches a value of ~ 8000, while the present linearly increases to that value, from which both start decreasing. Such decrease also occurs in the past oak model, opposite to the present one, which shows an increasing trend with radiation seasonality.

Last, regarding humidity, all the models show a constant suitability of the species until they reach ~ 5 g kg^−1^, where they show a quick increase, except for the *Acacia* 1963–1993 model, which remains constant at high probability of occurrence. The 1963–1993 oak model will increase the suitability and remain constant from this point. In contrast, both 1993–2023 models will result in an exponential decay as the humidity increases from this point on.

In this research, only the climatic variables were considered for the assessment of the species’ potential distribution. Thus, the potential competition for limited resources was not directly considered; instead, the climatic niche for both was evaluated and compared. Temperature was the main factor influencing the distribution probability of both taxa. Temperature is known to influence the reproductive mechanisms of plant species (Bykova et al., 2012), their growth and growing season length and physiological thresholds (Mod et al., 2016). Air humidity, on the other hand, has been linked to distribution of woody plants (Wang et al., 2022). For *A. dealbata* and *Q. robur*, our distribution model showed that even if not exactly equal, the niches of these species regarding these variables overlap to a large extent, especially in southern Europe. Regarding temperature, they both accept a similar range, but their averages differ by around 5 °C (14 °C for *Acacia* and 9 °C for oaks). *Quercus robur* is more adapted to continental climate than *Acacia*, which explains the greater spread northwards of oaks in the NH, where these colder climate types can be found.

### Niche and climatic profile overlap

Schoener’s D of the predictions of both species was 0.26 for the period between 1963 and 1993. It rose to 0.34 for the 1993–2023 period. Hellinger’s I was respectively 0.55 and 0.61 for each period. The major overlap hotspot is western Europe, especially on northern Spain and France (Fig. S3). Other hotspots include the south-eastern Australian coast and New Zealand, and some areas in the Pacific coast of America. Despite the increase in the overlap indices, the spatial distribution does not change much in time; it becomes more concentrated in the areas where it was already occurring.

Despite sharing a total area of 9.90 10^6^ km^2^ and mostly on the mid-latitudes (1993–2023 period), the climatic profiles for each taxon were significantly different for almost all climatic variables. The only variables showing no significant differences in their means (*p*-value > 0.1) were annual accumulated and monthly maximum precipitation (Table 2). *Acacia* appears in a wider amplitude of temperature daily range than oak. Similarly, *A. dealbata* shows a greater tolerance to contrasting precipitation regimes than *Q. robur*, tolerating a greater minimum monthly precipitation and precipitation seasonality (Table 2).

**Table 2:** Statistical summary and test-t results of the climatic variables in the areas where species occur, for the 1993–2023 period

Variable	Statistic	*A. dealbata*		*Q. robur*	
		Mean ± SD	[Min, max]	Mean ± SD	[Min, max]
Temperature	Average (°C)	13.59 ± 3.08	[4.21, 25.26]	8.62*** ± 2.98	[1.99, 18.69]
	Daily range (°C)	8.71 ± 3.52	[1.76, 18.01]	7.51*** ± 2.15	[2.19, 11.79]
	Isothermality	37.72 ± 10.45	[10.38, 89.11]	28.49*** ± 5.42	[14.07, 47.00]
	Seasonality	493.95 ± 155.07	[33.96, 908.63]	716.38*** ± 231.44	[317.29, 1311.95]
	Annual range (°C)	22.32 ± 6.65	[6.89, 42.23]	26.34*** ± 7.58	[11.60, 54.08]
Precipitation	Annual accumulated (mm)	731.69 ± 378.42	[111.46, 3629.02]	732.49 ± 168.30	[227.91, 1675.77]
	Maximum (month) (mm)	107.07 ± 58.39	[27.64, 437.68]	103.19 ± 30.58	[27.49, 243.48]
	Minimum (month) (mm)	26.45 ± 22.19	[0.30, 227.24]	35.40*** ± 11.23	[7.73, 68.47]
	Seasonality	46.32 ± 23.62	[13.97, 128.87]	36.25*** ± 14.14	[10.76, 81.44]
Solar radiation	Average (W m^−2^)	220.08 ± 24.73	[156.22, 289.36]	179.62*** ± 21.47	[131.73, 254.04]
	Annual range (W m^−2^)	223.47 ± 42.49	[24.04, 288.46]	257.91*** ± 16.59	[155.34, 297.08]
	Seasonality (10^3^)	8.22 ± 1.61	[0.764, 1.07]	9.57*** ± 0.64	[5.58, 11.14]
Specific humidity	Average (g kg ^−1^)	8.18 ± 1.60	[4.45, 18.60]	6.71*** ± 0.87	[5.03, 11.30]
	Annual range (g kg ^−1^)	5.25 ± 2.21	[1.03, 14.25]	6.46*** ± 1.70	[2.24, 12.72]
	Seasonality	194.89 ± 84.85	[35.68, 562.12]	239.37*** ± 61.94	[78.17, 471.01]

^***^p-value < 0.01

^**^0.01 < *p*-value < 0.05

^*^0.05 < *p*-value < 0.1

Regarding the most influential variables common to all the MaxEnt models, average temperature and average specific humidity were significantly different, with a significance level of 0.01. *Acacia* is adapted to warmer climates, with a preference for mild temperatures (13.6 °C) ranging between 4.2 and 25.3 °C; in comparison, oak shows a preference for colder climates (8.6 °C), in a range from 2.0 to 18.7 °C. Regarding the humidity, *Acacia* also shows a preference for higher value than *Q. robur*, the former with 8.2 g kg^−1^, and the latter with 6.7 g kg^−1^. *Acacia* appears in a wider average specific humidity amplitude, ranging from 4.4 to 18.6 g kg^−1^, while oak occurs in a range covering from 5.0 to 11.3 g kg^−1^.

### Evolution of potential distributions

**Fig. 3:**
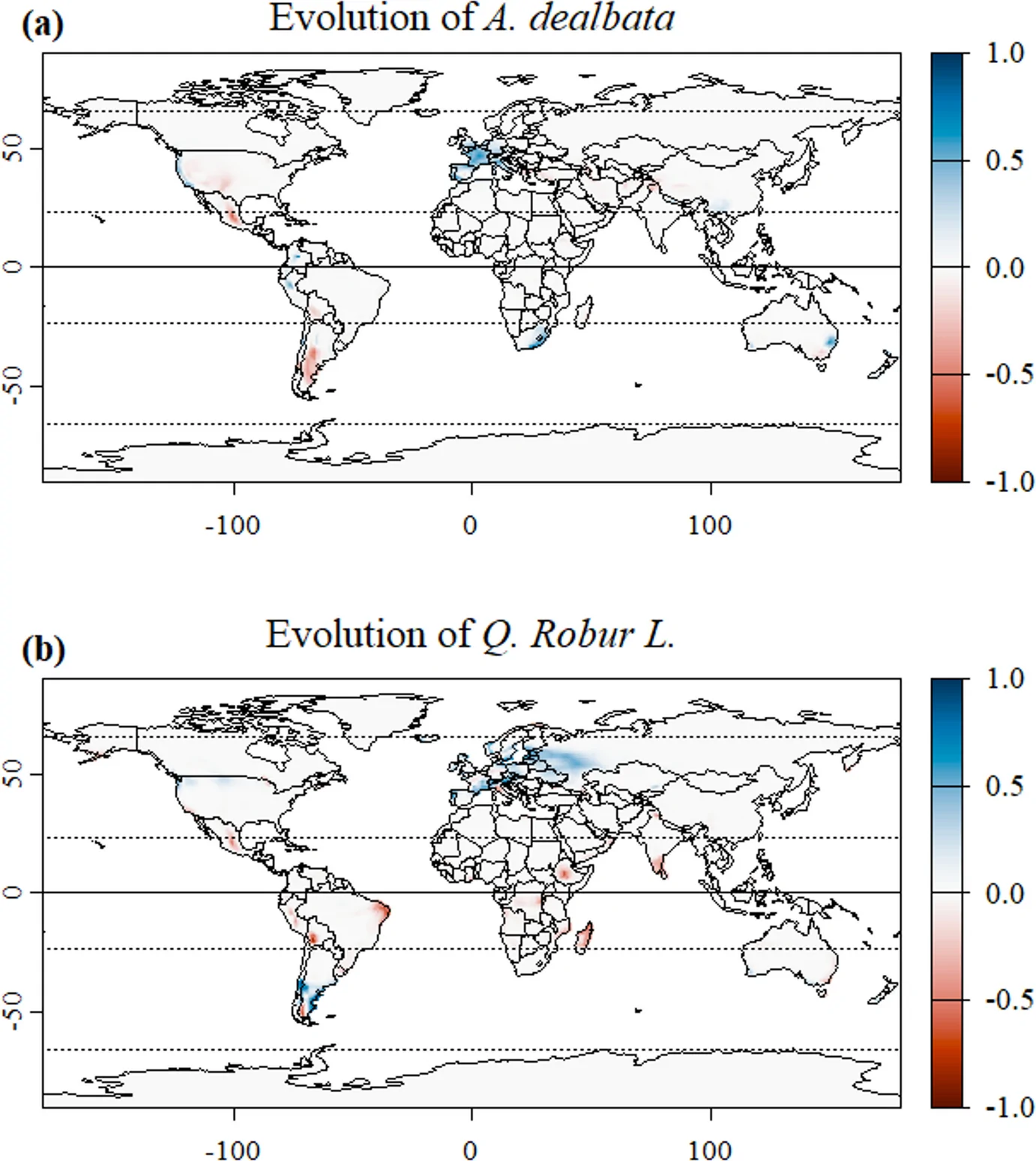
Differences in the modelled probabilities of favourable conditions for species between the periods standing for the present stage (1993–2023) and the previous (1963–1993). Species are **a** *A. dealbata* and **b** *Q. robur*

According to the MaxEnt models, *Acacia* suitability has expanded globally by 13.32% from the period 1963–1993 to 1993–2023 (Fig. 3). It has mostly extended in Europe, gaining some territory eastwards of its original distribution, reaching central Europe. Another major hotspot of *Acacia* spread is the western coast of North America, at latitudes similar to those where it has been invading Europe (~ 40°N). The overall shifts in *Acacia* suitability in the American continent are of 0.01, implying that the mean probability of occurrence of *Acacia* in the continent has not changed from one period to another. However, there are several changes in the distribution of niche suitability of *Acacia* within the continent (Fig. 3). Especially in Chile, the probability of occurrence of *Acacia* has mostly increased. On the other hand, in Brazil, it has dropped, specifically at the southern part of the country. Moreover, suitability of *Acacia* in South Africa has increased in the last period. In Oceania, where this species originates, its probability of occurrence has increased in the south-eastern Australia, although the overall global evolution in Australia remains close to 0. On the other hand, probability of occurrence for *Q. robur* has increased in western Asia and some parts of Mediterranean Europe, as well as in mid-latitude in South America. Central and South America, as well as some areas of the African continent registered an overall decrease of *Q. robur*, particularly in subtropical areas*.*

### Future scenarios

The models trained for both periods were ensembled and applied to climatic projections, namely the SSP1-2.6, SSP-2–4.5, SSP3-7.0, and SSP5-8.5. All the projections show an increase in the suitability of *A. dealbata* in the subtropical latitudes of the SH, particularly on the Pacific coast of South America. Similarly, in the mid-latitudes of the NH, there is a high suitability for *Acacia* on the North American Pacific coast, which decreases with the radiative forcing. This is not the case for Europe, where the species finds its climatic niche displaced towards the north with greater forcing. Thus, the suitability of *Acacia* is very limited to some Atlantic coasts on south-western Europe in the SSP1-2.6 scenario, but increases for the SSP2-4.5 and SSP3-7.0. Particularly in the latter, the suitability of *Acacia* in Mediterranean Europe is greatly increased. This does not happen in the SSP5-8.5, where the most suitable climatic niche for the species is found northwards from its current distribution, reaching the British Isles, especially the Irish Atlantic coast. Finally, there is an area bordering the Himalayan chain where the suitability increases with the radiative forcing.

The probability of niche suitability of *Q. robur* shows a similar behaviour depending on the radiative forcing, but with a preference for colder climates. Just like for *Acacia*, suitable niches exist in the American continent, both in the SH and in the NH. While for *Acacia* the general trend was to decrease with radiative forcing, *Q. robur* rather gets displaced towards the poles. However, it depicts a slightly different behaviour in Europe, where the suitability occurs mostly in the mid-latitudes, close to the central European coast, both in the SSP1-2.6 and the SSP2-4.5, with higher dispersion in the former, and higher concentration in the latter. In SSP3-7.0 scenario, the suitability of *Q. robur* generally increases in all of Europe, even reaching the Baltic areas and spreading eastwards. In this scenario, there also exists a suitable climate niche bordering the Himalayan chain (just like for *Acacia*). For this species, the probability of distribution in the SSP3-7.0 scenario is the closest to the current probability. In comparison, the niche suitability of *Q. robur* under scenario SSP5-8.5 concentrates further north than its original distribution, concentrating in the Scandinavian Atlantic coasts and the Irish and Scottish northernmost territories.

The niche overlap generally increased with the radiative forcing (Table 3). For most of the scenarios, the niche overlap occurs very strongly in the Pacific coast of South America, particularly in the mid-latitudes and in the Andes chain. This contrasts with the historical results, in which the niche overlap occurred more strongly in Europe. For scenarios SSP1-2.6 and SSP2-4.5 the overlapping in Europe is weaker than in the mentioned South American regions. In SSP3-7.0, however, the overlap predicted for the Andes is as strong as that for the European Atlantic coast. The SSP5-8.5 resulted in the strongest niche overlap (Table 3), which is mostly concentrated on north-western Europe and the mid-latitude Pacific coast of South America.

**Table 3:** Niche overlap indices for *A*. *dealbata* and *Q. robur* on each period and climatic projection

Period	Schoener’s D	Hellinger’s I
1963–1993 (historical)	0.2627	0.5569
1993–2023 (historical)	0.3408	0.6105
2024–2053 (SSP1-2.6)	0.3800	0.6936
2024–2053 (SSP2-4.5)	0.3999	0.6987
2024–2053 (SSP3-7.0)	0.4069	0.7046
2024–2053 (SSP5-8.5)	0.4485	0.7239

## Discussion

The main finding of this study shows that the suitable niche of *A. dealbata* is expanding polewards under climate change conditions, particularly associated with increasing temperatures and the shift of temperate zones towards Mediterranean climate (Alessandri et al., 2014). This expansion could increase the niche overlap between *A*. *dealbata* and temperate species, potentially increasing the areas where climatic conditions are suitable for both species and, consequently, the potential risk of future invasion. In this work, we use *Q*. *robur* as a representative temperate species, comparing the changes on its potential distribution relative to the potential distribution of *A*. *dealbata* under contrasting climate change scenarios. Our findings suggest that the niche overlap between the two species is increasing, but the extent it will reach in the future depends on the strength of climate forcing.

### Evolution of the species potential distribution

*Acacia* benefits from warmer climates, tolerating higher average temperatures. Temperature was one of the most influential variables in the *Acacia* models, suggesting that warmer climatic conditions may favour its potential distribution, relative to *Q. robur*. In fact, that would explain the northward expansion of *Acacia* suitability in Europe (increase of *Acacia* probability is greater than 60% in the Mediterranean areas), which could be associated with NH sensitivity to temperature shifts under climate change (Flato & Boer, 2001). Such a difference also could explain why shifts in the potential distribution of both taxa are of greater extent in the NH than in the SH, mostly concentrated in the Eurasian continent (Fig. 3). Also, the contribution importance of minimum precipitation was significantly higher in the 1963–1993 period than in the 1993–2023 (Fig. 1). The increase in precipitation in mid-latitudes (Dore, 2005) could have reduced the importance of water availability as a model constrain, potentially increasing the relative importance of temperature in the modelling of the species suitability. The modelled changes in niche suitability, particularly the expansion northwards in the NH (Fig. 3), are consistent with a growing influence of warmer temperatures. Determining factors that were not considered in this study are the soil characteristics and human action (afforestation, deforestation, elimination or introduction of the species to new areas, etc.).

With strategies such as efficient resource acquisition, allelopathic leachates, and pyrophytic traits, *A. dealbata* arises as a highly effective competitor (Morris et al., 2011). *Quercus*, on the other hand, has been enlisted as the dominant species of temperate broadleaf deciduous forests (Savill, 2004). The fact that these species share a climatic niche does not automatically make them direct competitors; however, understanding how their niche overlap evolves differently under different radiative forcing scenarios sheds light on the temperate areas that are prone to shifts in their vegetation structure due to the combined effects of climate change and the invasiveness of *Acacia*. Warmer summers and precipitation loss have already led to an increase in the drought frequency and intensity of Mediterranean areas, which is not only harmful for the ecosystem itself, but also increases the fire risk (MedECC, 2019). Fire risk can be increased by the presence of *Acacia* (Souza-Alonso et al., 2017), exacerbating the pressure on local biodiversity. Our results indicate that increasingly severe climate change scenarios are associated with the poleward expansion of the climatic niche of *Acacia*. These results suggest that new areas could become potentially sensitive to invasion, which, combined with climate change itself, could pose a threat to local biodiversity.

### Implications for management

The results presented in this work highlight the importance of proactive management to prevent and mitigate the effects of biological invasions. Our projections indicate that *A. dealbata* may find suitable conditions polewards of its current distribution (Fig. 3). This, given its invasive potential and ecological impacts, could pose a threat to the local biodiversity of the areas it reaches (Vieites-Blanco & González-Prieto, 2020). There exists reactive regulation in the areas where *Acacia* invasion is already an issue, including the ban for cultivation in Spain (MITECO, 2007) and Portugal (Ambiente e Transição Energética, 2019) or the heavy restrictions in South Africa (Department of Environment Forestry and Fisheries, 2020), California (California Department of Food & Agriculture, 2025), and the out-of-native-range Australia (Bellard et al., 2013). However, preventive measures should be prioritised in the regions that may become vulnerable to invasion in the future. This is the case of some European countries, where *A. dealbata* is cultivated as an ornamental plant, such as Croatia (Giovanetti et al., 2014), the UK, France, or Italy (Marchante et al., 2023). Our results show that some regions of the European Atlantic coast could be exposed to an increasing suitability for *A. dealbata* due to climate change, with France and the British Isles being potentially more affected with the increment of temperature. While the invasiveness of *A. dealbata* is currently recognised in France, the cultivation of the species has not been banned in the country due to its use in the cut-flower and perfume industries (Marchante et al., 2023). As opposed, in the British Isles, the species is cultivated mostly with ornamental purposes but its establishment in the wild is rather rare (Marchante et al., 2023). According to our results, however, this configuration could change in the future, especially under the more severe climate forcing scenarios. The climatic suitability of *A*. *dealbata* could increase in these countries. Although this alone does not directly imply the naturalisation and subsequent invasion of the species, it could increase the likelihood of establishment. Our results support the consideration of stricter regulation regarding the species in the potential invasion hotspots, although acknowledging that preventively banning the cultivation of the species could be deemed excessive. Instead, the implementation of early surveillance programmes to detect establishment and early stages of invasion is proposed as a valuable, less drastic strategy to prevent invasion (Langdon et al., 2019). In this regard, *A. dealbata* has been proven to be identifiable by remote sensing and state-of-the-art machine learning algorithms (Pinto et al., 2023), representing a cost-effective method for operational monitoring (Gwate et al., 2023). Particular attention is advised for riparian ecosystems, also for field-assessment, as *A. dealbata* has been found to gather around them (Zamorano et al., 2022). Furthermore, rapid response programmes ought to be designed to prevent the species from expanding and invading uninvaded areas that may become sensitive in the future due to climate change (Venette et al., 2021). In summary, invasion management, and especially prevention strategies, should account not only for the current distribution of *A. dealbata*, but also for projected future climatic suitability and potential invasion hotspots (Tataridas et al., 2022). The results presented in this work could aid in identifying the potentially sensitive areas to *A. dealbata* naturalisation and invasion from a climatic perspective, and therefore where the monitoring efforts and potential subsequent restrictions should be placed.

### Limitations and general discussion

We applied an SDM-based methodology whose results predict the potential distribution of species. We used an approach based on MaxEnt, which has proven to be a robust method for species distribution modelling based on climate niche suitability, but does have some limitations as well. First, the findings of this study depend on the reliability of the collected data of the species observations, as its broad nature (the study has been carried worldwide) would make it impossible to check presence or absence in the field. To account for a potential sampling bias, we developed the background points weighed by a global accessibility layer used as bias file. Additionally, the results of this model are influenced by the bias in sampling, as it only uses presence data (Phillips et al., 2006). To decrease the computational demand for obtaining the climatic statistics, we used a low-resolution grid and then interpolated it to a fourfold finer resolution. However, other factors that may shape the synoptic scale climatology were not considered, likely creating a smoother gradient in the variables. This may generate larger suitable areas than exist and artificially inflate AUC values. The generation of the background points is key in the smoothing of this. The definition of the MaxEnt framework itself implies another limitation, as it considers species to spread to a state of equilibrium in which they reach the maximum area that the considered conditions would allow (Phillips et al., 2006). However, there are some extra conditions, such as the soil characteristics, that have not been considered in this study. Our approach considered only the climatology as environmental factors affecting the potential distribution of species. Nonetheless, the conditions of land use, human practices, or other socioeconomic factors may and do influence in the potential distribution of *A. dealbata*. For example, the nitrification and acidification of soils due to nearby livestock-related activities (Liodakis et al., 2012) can favour the spread of such species instead of native ones (Lazzaro et al., 2014). Also, the spread of forest fires favours and is favoured by the presence of *A. dealbata* (Lorenzo et al., 2010), which creates a positive feedback loop that promotes invasion of such species. Moreover, MaxEnt model does not consider the biotic interactions (Phillips et al., 2006). We used a “control” species (*Q. robur*) which is native to some areas where *A. dealbata* could become highly invasive, but the model did not implement the potential interactions between them. Furthermore, the fact that two species share a climatic profile does not necessarily make them susceptible to direct competition. However, there is an extensive literature on the effects that Australian acacias have on temperate rainforests (Vieites-blanco & González-prieto, 2020). Last, the MaxEnt model cannot distinguish between the already suitable areas that have not been reached by the species and the newly suitable areas due to climate change scenarios, which could likely affect the discussion.

Despite all the aforementioned limitations, MaxEnt is a widely used and well-established tool for species distribution modelling. This approach was suitable for fulfilling the objectives of this research. Here, we modelled the niche suitability of *A. dealbata*, allowing us to identify potential hotspots of *A. dealbata* invasion in temperate forests; predicted the changes in the species' potential distribution due to climate change; and discussed the implications this has to the extent of area they share with other species (i.e. *Q. robur*). For the given species, we spotted the areas where their modelled climatic suitability is likely to converge. This is intended to be helpful in the decision-making process for biodiversity conservation by identifying areas where future co-occurrence is climatically possible. Thereafter, future works will consist of the implementation of biotic interactions in a model at a more regional scale. Also, the model here developed could be refined by implementing a larger group of variables as inputs such as soil characteristics and land use.

## Conclusions

In this study, several species distribution models were developed to understand the current, past, and future spread of *A. dealbata* in temperate forests. The models generally showed accurate performance with AUCs larger than 0.90. *A. dealbata* was predicted to occur with higher probability in the mid-latitudes, while extending into subtropical regions. On the other hand, Common European oak (*Q. robur*), modelled as a control species, occurs with higher probability in the mid-latitudes as well, but extending polewards more than equatorward. Both species occur simultaneously in southern and western Europe with high probability, which defines this area as a hotspot of niche overlap. Other overlapping hotspots are the west coast of North America, some regions of South America, South Africa, the southern coasts of Australia, and New Zealand. These areas fulfil the climatic niches for both taxa, regarding annual average temperature and humidity, which were the variables contributing most strongly for their distributions, according to the models. *Acacia* finds its suitable temperature at 13.6 °C and humidity at 8.2 g kg^−1^; on its part, the values for oak were 8.6 °C and 6.7 g kg^−1^. Thus, despite climate change favouring the invasion by *Acacia* of new areas where only oak occurs naturally, the probability of occurrence of *Q. robur* did not linearly decrease with radiative forcing in such areas. However, at mid-latitudes, where temperatures become increasingly unsuitable for *Q. robur* to thrive, its suitability is negatively affected under the climatic scenarios. On the other hand, the reasons why *Acacia* suitability decreases in some areas (despite its general ability to spread and invade) remain unknown. Climate change-induced shifts in weather parameters do not seem to be the cause. Neither does human intervention. Thus, deeper research into this issue is warranted, as it is of great interest to understand the mechanisms that can lead to a reduction of *Acacia*, considered invasive in several regions worldwide. This work contributes to identifying the global-scale perspective of potential co-occurrence areas under contrasting climate change scenarios, whose effects for local biodiversity may be exacerbated by *Acacia* invasions. To the best of our knowledge, this subject, particularly at a global scale, has not yet been widely addressed.

## Supplementary Information

Below is the link to the electronic supplementary material.

ESM 1 (PNG 1.51 MB)

High Resolution Image (TIFF 2.57 MB)

ESM 2 (PNG 1.57 MB)

High Resolution Image (TIFF 2.57 MB)

ESM 3 (PNG 1.50 MB)

High Resolution Image (TIFF 2.57 MB)

ESM 4 (DOCX 19.6 KB)

## Data Availability

Historical climate data was retrieved from NOAA PSL at https://psl.noaa.gov/, climatic projections from CESM2 were obtained from Copernicus CDS at https://cds.climate.copernicus.eu/ and occurrence data for the species was provided by GBIF at https://www.gbif.org/. The results obtained on this research can be found at Zenodo, at https://zenodo.org/records/14671257
