# Supplementary material for: Projected climate change could expand the geographical suitability of Acacia dealbata into new temperate areas
Source: Environ Monit Assess. 2026 Sep 29;198(10):1131. doi: 10.1007/s10661-026-15951-5 (PMC13623992; doi:10.1007/s10661-026-15951-5)
Supplement: Supplementary file 1 [file 10661_2026_15951_MOESM4_ESM.docx]

**Table 1:** Reference studies in which Species Distribution Models were used to predict the distribution of some species of the genus *Acacia.*

| **Reference** | **Area** | **Methodology *** | **Results **** | **Species** |
| --- | --- | --- | --- | --- |
| César de Sá et al. (2019) | Portugal | Ensemble | [0.88, 0.92]  [0.94, 0.95]  [0.90, 0.96] | *A. dealbata*  *A. longifolia*  *A. melanoxylon* |
| Fernandes et al. (2019) | NW Iberian Peninsula | Ensemble | [0.86, 0.95] | *A. dealbata* |
| Fernandes et al. (2014) | NW Portugal | Ensemble | [0.94, 0.97] | *A. dealbata* |
| Dinis et al. (2020) | South Africa, Portugal & SE Australia | Ensemble | [0.79, 0.94]  [0.71, 0.92] | *A. longifolia*  *T. acaciaelongifoliae* |
| Monteiro et al. (2017) | Serra do Gerês (Portugal) | RF | acc: 91%  κ: 0.81 | *A. dealbata* |
| Costa et al. (2013) | Açores (Portugal) | ENFA | - | *P. undulatum*  *A. melanoxylon* |
| Vicente et al. (2013) | N Portugal | Ensemble | - | *A. dealbata*  *A. longifolia*  *A. melanoxylon* |
| Langdon et al. (2019) | SC Chile | MaxEnt | Global: 0.95  Invaded: 0.83  Native: 0.81 | *A. dealbata* |
| Bustamante et al. (2022) | Chile | MaxEnt | [0.92, 0.94]  [0.91, 0.96] | *A. dealbata*  *A. melanoxylon* |
| Silva et al. (2023) | Chile | RF | 0.957  0.968 | *A. dealbata*  *A. melanoxylon* |
| Hirsch et al. (2017) | South Africa | Ensemble | [0.97, 0.99]  TSS > 0.9 | *A. dealbata* |
| Thompson et al. (2011) | South Africa | MaxEnt | - | *A. saligna* |
| Lozano et al. (2020) | Sardinia (Italy) | GLM | 0.94 | *A. saligna* |
| El-Barougy et al. (2021) | Egypt | TrEnv | 0.78 | *A. saligna* |
| Heringer et al. (2019) | Brazilian Atlantic Forest | MaxEnt | train: [0.82, 0.95]  test: [0.91, 0.93]  train: [0.94, 0.97]  test: [0.95, 0.96] | *A. mangium*  *A. auriculiformis* |
| Higgins et al. (2020) | Global | Maxent  TTR-std  TTR-fqr | 0.98  0.95  0.95 | *A. saligna* |

*Ensemble stands for a methodology in which the outputs of several models are combined (see Araújo & New (2007)). Other options are RF (Random Forest), ENFA (Ecological-Niche Factor Analysis), MaxEnt (Maximal Entropy), GLM (Generalised Linear Models), TrEnv (Trait-Environment), TTR-std (standard Thornley Transport Resistance) and TTR-fqr (Farquhar Thornley transport resistance).

**Results are expressed in terms of Area Under Receiver Operator Curve (AUC, ROC), unless otherwise specified. Alternate options are: acc (accuracy); κ (Kohen’s Kappa) or TSS (True Skill Score).
